# The effect of a voucher incentive on a survey response rate in the clinical setting: a quasi-randomized controlled trial

**DOI:** 10.1186/s12874-018-0544-4

**Published:** 2018-08-16

**Authors:** Dawid Pieper, Nina Kotte, Peggy Ober

**Affiliations:** Institute for Research in Operative Medicine, Chair of Surgical Research, Faculty of Health, School of Medicine, Witten/Herdecke University, Ostmerheimer Str, 200 51109 Cologne, Germany

**Keywords:** Motivation, Outcome assessment, Questionnaire, Randomized controlled trial, Response rate

## Abstract

**Background:**

Financial rewards have been shown to be an important motivator to include normal healthy volunteers in trials. Less emphasis has been put on non-healthy volunteers. No previous study has investigated the impact of a voucher incentive for participants in a cross-sectional study in a clinical setting. The objective of this study was to examine the impact of a small voucher incentive on a survey response rate in a clinical setting at the point-of-care in a quasi-randomized controlled trial (q-RCT).

**Methods:**

This was an ancillary study to a survey of patients subsequent to their appointment with a physician investigating physician-patient communication. We randomized participants to receive or not receive a voucher for a coffee (costs: 1 €) enclosed in the survey package. Alternation of groups was performed on a weekly basis. The exact Chi-square test was used to compare response rates between study arms.

**Results:**

In total, 472 participants received the survey package. Among them, 249 participants were quasi-randomized to the voucher arm and 223 to the control group. The total response rate was 46%. The response rates were 48% in the voucher arm and 44% in the control group. The corresponding risk ratio was 1.09 (95% CI: 0.89, 1.32).

**Conclusions:**

A small voucher incentive to increase the response rate in a survey investigating physician-patient communication was unlikely to have an impact. It can be speculated whether the magnitude of the voucher was too low to generate an impact. This should be further investigated in future real-world studies.

## Background

Recruitment can be defined as “the dialogue which takes place between an investigator and a potential participant prior to the initiation of the consent process” [1]. Recruitment of study participants poses a challenge to research studies. Poor recruitment can result in underpowered studies. Low response rates can introduce bias and reduce certainty in the study results [2–4]. In the worst case, studies might be stopped or abandoned. One study found that only 31% of trials achieved their original recruitment target, while 53% needed to be extended [5].

Problems with recruitment are dependent on the study type. In general, recruitment problems are more likely in clinical trials and cohort studies. Recruitment in cross-sectional studies with only one time point of data collection should generally be less difficult. However, there is a paucity of literature on this topic, making it difficult to underpin this statement with empirical results. Therefore, it is not surprising that the majority of studies investigating strategies to increase recruitment have focused on clinical trials [6].

Among other methods, incentive-based approaches such as small gifts, vouchers or financial incentives have been investigated [6, 7]. These approaches are often used to facilitate study participation among persons who might otherwise not participate [8]. Financial rewards were also shown to be an important motivator for including normal healthy volunteers in trials [9]. The character and magnitude of the voucher can also have an impact on its effectiveness [10].

To the best of our knowledge, no previous study has investigated the impact of a voucher incentive for participants in a cross-sectional study in a clinical setting. Against the background that hypothetical studies to investigate recruitment strategies have their place in this field of research, researchers are encouraged to include an evaluation of recruitment interventions within their studies [6]. Therefore, we conducted a quasi-randomized controlled trial (q-RCT) to examine the impact of a small voucher incentive on a survey response rate in a clinical setting at the point-of-care.

## Methods

We conducted the q-RCT as an ancillary study to a survey of patients subsequent to their appointment with a physician [11]. All appointments were held during surgery hours. The aim of the survey was to investigate physician-patient communication.

### Trial design

We randomized patients per their week of appointment at the clinic to one of two groups: a) the voucher arm that consisted of a survey package with an enclosed voucher for a coffee (costs: 1 €) to redeem at one of the three cafeterias of the hospital; b) the no voucher arm that only consisted of a survey package without a voucher. Alternation of groups was performed on a weekly basis (i.e. patients attending the clinic in odd weeks received the voucher, while patients attending the clinic in even weeks did not). The survey package consisted of a cover letter, an information leaflet, the questionnaire and a franked return envelope to send back the questionnaire. The voucher was affixed to the top of the questionnaire. In addition, study assistants referred to the voucher when handing out the survey package.

### Study population

The study population consisted of outpatients attending surgical hours at a university-affiliated hospital in Cologne, Germany. Participating surgeons had to give informed consent. Eleven surgeons from four departments agreed to participate. Only persons attending a participating surgeon were eligible for inclusion into the study. There were no other inclusion criteria for the participants.

The ethical review board of Witten/Herdecke University approved the study and waived the requirement for a signed consent form. Participants received an information leaflet about the survey’s aim including similar information usually included in a consent form.

### Survey design

The Individual Clinician Feedback (ICF) instrument developed by PICKER Europe in 2012 was used for the survey. The aim of the ICF is to collect feedback on physicians’ communication skills based on patients’ experience during their appointment. The questionnaire was translated into German and culturally adapted using established methods [12]. The German ICF questionnaire consists of 38 items with responses ranging from 0 to 10 on a Likert scale, with higher values indicating a higher satisfaction.

Study assistants were present during surgery hours of thoracic surgery, general surgery, orthopedics and trauma surgery, and plastic surgery. Potential participants in the study were approached by study assistants immediately after the appointment. Thus, there was no possibility for sending reminders as no contact information was collected. Study assistants were not blinded (i.e. they were aware of whether the survey package contained the voucher). A franked return envelope was included in the survey package to maximize response rate. The survey took place from June to August 2015 (13 weeks). Data collection was restricted to this time period in this pilot study. As this was a pilot study, and we were also interested to investigate population volume, no sample size calculation was performed a priori.

### Statistical analysis

The primary outcome was the response rate. We defined the response rate as the number of completed questionnaires divided by the number of delivered questionnaires. The exact Chi-square test was used to compare response rates between study arms. We considered two-sided *p* values and 0.05 for statistical significance.

## Results

In total, 472 participants received the survey package. Among them, 249 participants were quasi-randomized to the voucher arm and 223 to the control group. The total response rate was 46%. The response rates were 48% (120/249) in the voucher arm and 44% (98/223) in the control group. The difference of 4% points was statistically not significant (*p* = 0.623). The corresponding risk ratio was 1.09 (95% CI: 0.89, 1.32). The risk ratios ranged from 0.99 to 1.80 across the four different surgery hours, none of the risk ratios being statistically significant.

## Discussion

We conducted a qRCT to investigate the impact of a small voucher incentive on a survey response rate of patients after their appointment with a physician. We found that this voucher incentive was unlikely to have had an impact on the response rate.

Our total response rate of 46% is lower than in other studies dealing with communication. Previous studies showed response rates varying between 74 and 83% in different settings, such as dentistry [13], hospital [14], cancer [15], or cardiovascular risk [16]. However, a very similar, but large-scale study found a response rate of 51% for a mailed questionnaire of patients’ experience of face-to-face consultations with general practitioners in the UK [17]. An older review found a mean response rate of approximately 60% among mail surveys published in medical journals [3].

There are not many studies we could compare our results with. Most focus on effective strategies for recruitment to trials. Trials are different from our study in that trials require a follow-up of patients. We did not follow up on the included patients (i.e. data collected at one time point only). Furthermore, patients included in trials mainly suffer from a disease or a health care problem under study, excepting prevention trials. Therefore, in the absence of comparable studies, we can probably best compare our study results with studies dealing with the recruitment of normal healthy volunteers. Our study sample did not consist of healthy volunteers only, as patients were attending surgery hours for an underlying health care problem. However, the focus of our study investigating physician-patient communication is not set on an underlying health care problem.

Ill patients might seek relief, cure or a better understanding of their condition, and this might promote their participation in a trial or a research study [18]. The benefits for healthy volunteers remain less clear [19], and it has been debated whether they can be motivated by financial or voucher incentives given that their motivation might arise from something different [20]. However, a systematic review found financial incentives to be a strong motivator for the participation of healthy volunteers in trials [9]. Similar results were also found for surveys. A recent study found that a £2.50 shop voucher significantly improved response rates when compared to no voucher (43% vs. 38%) [21]. This study is also of particular interest as another study group received a £5.00 shop voucher, but this did not have a significant effect compared to the £2.50 shop voucher group (42% vs. 43%). This study provides evidence that even small voucher incentives might be able to increase response rates. A £5.00 gift voucher, redeemable at a range of shops, was investigated as an incentive to improve the response rate of a postal questionnaire in a RCT [22]. More questionnaires were returned in the incentive arm (risk ratio 1.10 (95% CI 1.05, 1.16)) when compared to the no incentive group. A similar voucher resulted in a 11.7% (95% CI 4.7 to 18.6%) improvement in the response rate of mothers of seven-year-old children, where the latter were to be assessed [23]. However, the incentive used in our study was smaller than in any of the studies mentioned above. Therefore, the voucher might have been too small to have an impact.

Another difference of our study is that questionnaires were not mailed, but handed out personally by the study personnel. This is a rather uncommon strategy as it results in higher resources needed to conduct the study. Personalized questionnaires and letters as well as contacting participants before sending questionnaires were shown to increase response rates in a meta-analysis [24].

It has been stressed before that studies dealing with health communication research might be different with respect to recruitment of patients, among other aspects [25]. The reason for this is the context (i.e. the clinic) within which communication takes place. When studying physician-patient communication it also important to include the clinical staff. Shue 2004 has pointed out three main issues when recruiting participants in the clinical setting: researcher availability, clinical staff knowledge, and clinic scheduling constraints [25]. All of them were considered by us when planning and conducting the study.

We were unable to randomize vouchers in a truly randomized fashion, but performed a quasi-randomized trial instead. Alternation has the drawback that future assignments can be anticipated or are simply known, as in our study. Thus, selection bias might have occurred due to the selective enrolment and non-enrolment of participants into the study [26]. The choice for alternation was made as we could not rule out the possibility that patients sitting in the waiting room would see another patient being included into the study and randomized either to the voucher incentive arm or no incentive arm. This might have introduced the danger of decreasing the willingness of those patients to participate in the study who did not receive the voucher. As patients were asked to participate in the study subsequent to leaving the doctors examination room, it was not possible to randomize patients in another room for practical reasons. Only such a procedure would have made it possible to mask the randomization and not to interfere with other patients sitting in the waiting room.

A limitation of our study is the relatively small sample size, which was due to its nature as a pilot study. Similarly, as we have included a wide range of patients with different conditions, the generalizability of our study results might be limited. A strength of our study is that we report real-world results, while it has been criticized that many studies investigating strategies for increasing participation rates are hypothetical [6].

## Conclusions

A small voucher incentive to increase the response rate in a survey investigating physician-patient communication was unlikely to have an impact. It can be speculated whether the magnitude of the voucher was too low to generate an impact. This should be further investigated in future real-world studies. Different study designs, objectives and contexts (e.g. heath communication) should also be taken into consideration.

## Abbreviations

qRCT: quasi-randomized controlled trial
RCT: randomized controlled trial
RR: risk ratio
